# Isolation of a Genomic Region Affecting Most Components of Metabolic Syndrome in a Chromosome-16 Congenic Rat Model

**DOI:** 10.1371/journal.pone.0152708

**Published:** 2016-03-31

**Authors:** Lucie Šedová, Michal Pravenec, Drahomíra Křenová, Ludmila Kazdová, Václav Zídek, Michaela Krupková, František Liška, Vladimír Křen, Ondřej Šeda

**Affiliations:** 1 Institute of Biology and Medical Genetics, First Faculty of Medicine, Charles University and the General Teaching Hospital, Prague, Czech Republic; 2 Institute of Molecular Genetics of the Czech Academy of Sciences, Prague, Czech Republic; 3 Institute of Physiology of the Czech Academy of Sciences, Prague, Czech Republic; 4 Department of Metabolism and Diabetes, Institute for Clinical and Experimental Medicine, Prague, Czech Republic; University of Barcelona, Faculty of Biology, SPAIN

## Abstract

Metabolic syndrome is a highly prevalent human disease with substantial genomic and environmental components. Previous studies indicate the presence of significant genetic determinants of several features of metabolic syndrome on rat chromosome 16 (RNO16) and the syntenic regions of human genome. We derived the SHR.BN16 congenic strain by introgression of a limited RNO16 region from the Brown Norway congenic strain (BN-*Lx*) into the genomic background of the spontaneously hypertensive rat (SHR) strain. We compared the morphometric, metabolic, and hemodynamic profiles of adult male SHR and SHR.BN16 rats. We also compared *in silico* the DNA sequences for the differential segment in the BN-*Lx* and SHR parental strains. SHR.BN16 congenic rats had significantly lower weight, decreased concentrations of total triglycerides and cholesterol, and improved glucose tolerance compared with SHR rats. The concentrations of insulin, free fatty acids, and adiponectin were comparable between the two strains. SHR.BN16 rats had significantly lower systolic (18–28 mmHg difference) and diastolic (10–15 mmHg difference) blood pressure throughout the experiment (repeated-measures ANOVA, P < 0.001). The differential segment spans approximately 22 Mb of the telomeric part of the short arm of RNO16. The *in silico* analyses revealed over 1200 DNA variants between the BN-*Lx* and SHR genomes in the SHR.BN16 differential segment, 44 of which lead to missense mutations, and only eight of which (in *Asb14*, *Il17rd*, *Itih1*, *Syt15*, *Ercc6*, *RGD1564958*, *Tmem161a*, and *Gatad2a* genes) are predicted to be damaging to the protein product. Furthermore, a number of genes within the RNO16 differential segment associated with metabolic syndrome components in human studies showed polymorphisms between SHR and BN-*Lx* (including *Lpl*, *Nrg3*, *Pbx4*, *Cilp2*, and *Stab1*). Our novel congenic rat model demonstrates that a limited genomic region on RNO16 in the SHR significantly affects many of the features of metabolic syndrome.

## Introduction

Metabolic syndrome is defined as the clustering of several conditions in one individual, including obesity, hypertension, insulin resistance, and dyslipidemia. According to the consensus definition reached in 2009 by several major professional bodies [1], the presence of any three of the following criteria provides a basis for the clinical diagnosis of metabolic syndrome: waist circumference surpassing a geoethnic- and sex-specific threshold, systolic blood pressure ≥135 mmHg and/or diastolic blood pressure ≥85 mmHg (or treatment with antihypertensive therapy), triglyceride concentration ≥1.7 mmol/l, high-density lipoprotein cholesterol <1.0 or 1.3 mmol/l for men and women, respectively (or treatment with hypolipidemic therapy), and fasting glucose >5.6 mmol/l (or treatment with glucose-lowering therapy). Obesity, hypertension, insulin resistance, and dyslipidemia are all complex traits with substantial contributions from genomic and environmental factors, and their interaction [2]. Metabolic syndrome is thus a *meta*-complex trait. There is ample evidence both for genetically driven susceptibility to metabolic syndrome [3] and for the importance of lifestyle, epigenetic, and developmental factors [4]. It remains to be determined whether the hereditary component of metabolic syndrome is represented simply by a supercritical sum of risk alleles for the individual features of the syndrome or whether there are particular variants within perturbed metabolic and signaling networks that are specific for the transition to metabolic syndrome as a whole.

As with other multifactorial diseases, detailed analysis of the genomic component of metabolic syndrome within the general human population is complicated by numerous factors that cannot be easily controlled [5]. The promise that genome-wide association studies could elucidate the genetic architecture of metabolic syndrome is slowly fading away despite a number of reported significant associations. Currently, there are 1026 associations reaching genome-wide significance (P < 5 × 10^−8^) for individual components of metabolic syndrome, bivariate traits, or the complete metabolic syndrome, according to the NHGRI-EBI Catalog of Published Genome-Wide Association Studies (http://www.ebi.ac.uk/gwas, accessed on Nov 26^th^, 2015). As the systems genetic-level analysis of complex diseases is still at a nascent stage [6], reductionist models represent a feasible option for genetic dissection of metabolic syndrome [7, 8]. In particular, we foresee that comprehensive models with extensive biological annotation will be of major importance in the years to come.

The reference rat system for the genetics of metabolic syndrome is the HXB/BXH recombinant inbred (RI) panel [6, 9], derived from the spontaneously hypertensive rat (SHR) strain [10] and its normotensive counterpart, the Brown Norway BN-*Lx* congenic strain [11]. One of the regions showing consistent linkage to blood pressure in segregating populations derived from SHR and BN rats [12, 13], as well as other hypertensive strains [14–16], maps to the short arm of rat chromosome 16 (RNO16). However, it is always necessary to validate the physiological significance of identified loci, optimally in conjunction with a comparative genomic approach to establish the potential relevance of the tested variation for the human condition [17]. An established strategy for this is the construction of genetically designed congenic strains, in which the extracted locus acts on the genomic background of the counterpart rat strain. In the process of validating several quantitative trait loci (QTLs) for blood pressure identified in an F2 SHR/BN cross, Aneas et al. derived the SHR.BN-(D16Rat87-D16Mgh1)/Jk congenic strain. The transfer of an approximately 40 Mb BN segment into the SHR genomic background did not result in significant lowering of basal blood pressure; nevertheless, it prevented blood pressure from increasing on salt loading [13]. Moreover, RNO16 bears loci affecting other components of metabolic syndrome [18–22]. The aim of the current study was to validate and further explore the role of this QTL in hypertension and related metabolic disturbances, taking a comparative genomic approach.

## Materials and Methods

### Ethics statement

This project was performed in conformity with the Animal Protection Law of the Czech Republic. The experimental protocols and detailed procedures were evaluated and approved by the Ethical Committee of the First Faculty of Medicine, Charles University in Prague and by the Ministry of Education, Youth and Sports of the Czech Republic. The health of the rats was examined daily, and the animals were monitored every hour during the experimental procedures. There were no unexpected deaths during the experiment. Overdose of anesthetic (halothane) was the method of euthanasia in this study. All efforts were made to minimize suffering of the experimental animals.

### Derivation of the SHR.BN16 congenic strain

The SHR/OlaIpcv [SHR hereafter, Rat Genome Database (RGD) [23], http://rgd.mcw.edu, ID no. 631848] and BN-*Lx*/Cub (BN-*Lx* hereafter, RGD ID no. 61117) [11] strains were maintained at the Institute of Medical Biology and Genetics, Charles University in Prague. To derive the SHR.BN16 congenic strain, a marker-assisted backcross breeding approach was used, as described previously [11, 24, 25]. In short, SHR rats were crossed with BN-*Lx*/Cub rats and the subsequent F1 hybrids were repeatedly backcrossed to SHR/OlaIpcv. The differential segment was fixed by intercrossing heterozygotes and selecting the progeny with homozygous BN/Cub-derived chromosome 16 segments. The congenic status of the new SHR.BN16 strain was validated with a whole-genome marker scan.

### Experimental protocol

Adult male rats were housed under temperature- and humidity-controlled conditions with a 12 h/12 h light–dark cycle. Animals had free access to food (standard chow) and water at all times. At 4 months of age, males from the SHR.BN16 congenic strain (n = 10) and the parental SHR strain (n = 9) were subjected to an oral glucose tolerance test (OGTT) after overnight fasting and blood samples were drawn. The animals were then sacrificed, and their total weight and the weights of the heart, liver, kidneys, and adrenal glands, and the epididymal and retroperitoneal fat pads, were determined.

### DNA extraction and genotyping

Rat DNA was isolated from tail samples by the modified phenol extraction method. Primer nucleotide sequences were obtained from public databases [RGD (http://rgd.mcw.edu), the Wellcome Trust Centre for Human Genetics, (http://www.well.ox.ac.uk), and the Whitehead Institute/MIT Center for Genome Research (http://www-genome.wi.mit.edu)]. Polymerase chain reaction (PCR) was used for genotyping markers polymorphic between progenitor strains. We tested DNA from the congenic strain (SHR.BN16, n = 10) and the progenitor strains SHR and BN-*Lx*/Cub. The PCR products were separated on polyacrylamide (7–10%) gels and detected in UV light after ethidium bromide staining using Syngene G:Box (Synoptics, Ltd., Cambridge, UK).

### Metabolic measurements

The OGTT was performed after overnight fasting. Blood samples for glycemic assessment (Ascensia Elite Blood Glucose Meter, Bayer HealthCare, Mishawaka, IN, USA; validated by the Institute of Clinical Biochemistry and Laboratory Diagnostics of the First Faculty of Medicine, Charles University in Prague) were obtained from the tail vein at intervals of 0, 30, 60, 120, and 180 min after intragastric glucose administration to conscious rats (3 g/kg body weight, 30% aqueous solution). Serum triglyceride and cholesterol concentrations were measured by standard enzymatic methods (Erba-Lachema, Brno, Czech Republic). Serum free fatty-acid levels were measured with an acyl-CoA oxidase-based colorimetric kit (Roche Diagnostics GmbH, Mannheim, Germany). Enzyme-linked immunosorbent assay (ELISA) kits were used to determine the serum levels of insulin (Mercodia, Uppsala, Sweden) and adiponectin (BioSource, San Diego, CA, USA).

### Blood-pressure assessment

Blood-pressure assessment was performed on a separate group of animals than those used in the metabolic studies. Male SHR and SHR.BN16 rats (n = 8 per strain) were surgically implanted with radiotelemetry transducers under ketamine/xylazine anesthesia. After surgery, the rats received postoperative analgesia (buprenorphine) and were allowed to recover for at least 7 days. Starting at 10 weeks of age, arterial blood pressure was measured continuously by radiotelemetry in the conscious, unrestrained, rats for 41 days. Rats had free access to water and standard chow. Pulsatile pressure was recorded in 10 s bursts every 10 min throughout the day and night, and 12 h averages for systolic and diastolic arterial blood pressure were calculated for each rat over the 41 day recording period. For 5 days (from day 28 to day 32), rats were given 1% NaCl to drink instead of tap water.

### Statistical analysis

All statistical analyses were performed using STATISTICA 12 CZ. Unpaired Student’s t-tests were used to compare metabolic and morphometric traits in the SHR and SHR.BN16 strains. Repeated-measures analyses of variance (ANOVAs) were used to compare systolic and diastolic blood pressure. The null hypothesis was rejected whenever P < 0.05.

### In silico analyses

The Virtual Comparative Map software tool (http://www.animalgenome.org/VCmap) was used to identify the regions of the human genome syntenic to the differential segment in the SHR.BN16 congenic strain. These regions were then compared with the significant loci reported in human genome-wide association studies (extracted from the Catalog of Published Genome-Wide Association Studies, available at: http://www.ebi.ac.uk/gwas [26]). To compare the publically available DNA sequences of SHR/OlaIpcv and BN-*Lx*/Cub, we used the Variant Visualizer resource provided by the RGD (http://rgd.mcw.edu/rgdweb/front/select.html) with high conservation settings (0.75–1) determined by PHAST (http://compgen.cshl.edu/phast) [27], minimum read depth set to eight, and exclusion of variants found in fewer than 15% of reads. The results were then verified in the relevant NCBI-based databases.

## Results

### Genomic characterization of the SHR.BN16 congenic strain

**Fig 1:**
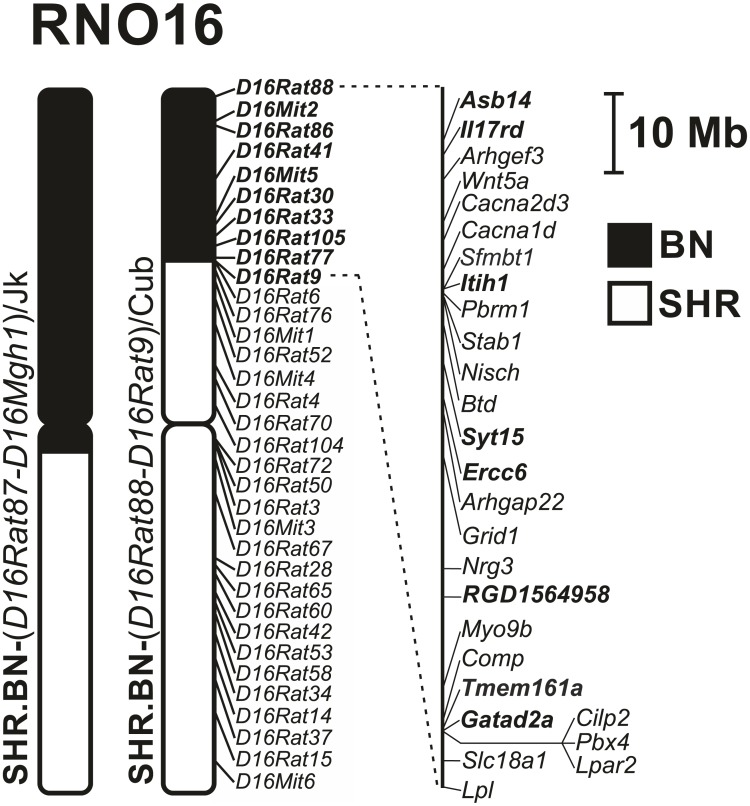
The rat chromosome 16 (RNO16) differential segment in the SHR.BN-(*D16Rat88-D16Rat9*)/Cub strain. The RNO16 differential segment in the SHR.BN-(*D16Rat88-D16Rat9*)/Cub strain compared with a previously published congenic strain carrying Brown-Norway-origin RNO16 (SHR.BN-(*D16Rat87-D16Mgh1*)/Jk) [13]. The RNO16 markers genotyped in this study to determine the differential segment in the SHR.BN-(*D16Rat88-D16Rat9*)/Cub strain are shown to the right of the chromosome. Chromosomal regions of SHR origin are depicted by open bars; the BN-derived segment is shown as a solid bar. Genes with “probably damaging” nonsynonymous mutations distinguishing SHR from BN-*Lx* are shown in **bold**. Genes that are associated with features of metabolic syndrome in human genome-wide association studies and that show variance between SHR and BN-*Lx* (*in silico* comparison) are shown in italics. *Asb14*—ankyrin repeat and SOCS box-containing 14, *Il17rd—*interleukin 17 receptor D, *Arhgef3—*Rho guanine nucleotide exchange factor 3, *Wnt5a -* wingless-type MMTV integration site family, member 5A, *Cacna2d3—*calcium channel, voltage-dependent, alpha2/delta subunit 3, *Cacna1d -* calcium channel, voltage-dependent, L type, alpha 1D subunit, *Sfmbt1—*Scm-like with four mbt domains 1, *Itih1—*inter-alpha-trypsin inhibitor heavy chain 1, *Pbrm1—*polybromo 1, *Stab1*—stabilin 1, *Nisch*—nischarin, *Btd*—biotinidase, *Syt15—*synaptotagmin XV, *Ercc6—*excision repair cross-complementing rodent repair deficiency, complementation group 6, *Arhgap22*—Rho GTPase activating protein 22, *Grid1—*glutamate receptor, ionotropic, delta 1, *Nrg3*—neuregulin 3, *RGD1564958*—similar to glyceraldehyde-3-phosphate dehydrogenase (phosphorylating) (EC 1.2.1.12), *Myo9b* (myosin IXb), *Comp*—cartilage oligomeric matrix protein, *Tmem161a* (transmembrane protein 161A), *Gatad2a -* GATA zinc finger domain containing 2A, *Cilp2*—cartilage intermediate layer protein 2, *Pbx4—*pre-B-cell leukemia homeobox 4, *Lpar2*—lysophosphatidic acid receptor 2, *Slc18a1*—solute carrier family 18 (vesicular monoamine transporter), member 1, *Lpl*—lipoprotein lipase.

We used a genotyping scan with a set of 34 markers polymorphic between SHR and BN-*Lx* on chromosome 16 to reveal the extent of the BN-*Lx*-origin differential segment in the SHR.BN-(*D16Rat88-D16Rat9*)/Cub congenic strain (SHR.BN16 hereafter). The differential segment spans about 22 Mb at the telomeric end of the RNO16 short arm (Fig 1). Several total genome scans conducted during derivation of the SHR.BN16 strain excluded the presence of non-SHR alleles other than those fixed on RNO16, confirming the congenic status of the new strain. The BN-*Lx*-derived RNO16 segment hence represents the only genomic difference between the SHR and SHR.BN16 strains.

### Metabolic comparison of the SHR and SHR.BN16 strains

**Fig 2:**
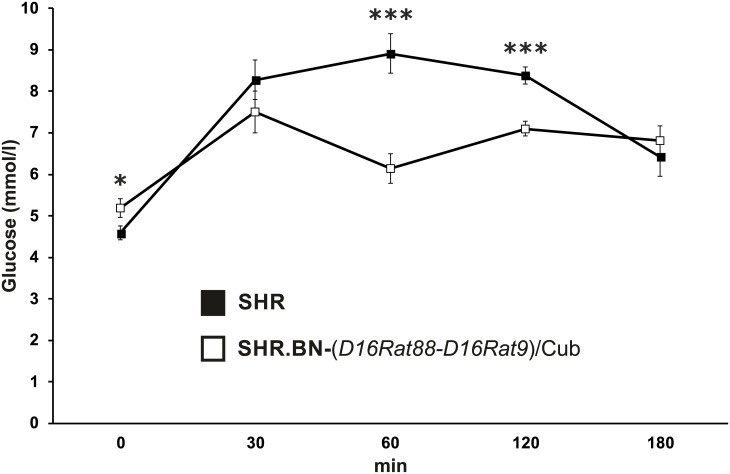
The oral glucose tolerance test in SHR and SHR.BN-(*D16Rat88-D16Rat9*)/Cub strains. Glycemic time courses for adult male SHR (black squares) and SHR.BN-(*D16Rat88-D16Rat9*)/Cub (white squares) rats fed a standard diet. * P < 0.05; *** P < 0.001.

Adult SHR males were about 10% heavier than adult SHR.BN16 males. The relative weight of the adipose tissue depots was decreased in the congenic strain, reaching statistical significance for the retroperitoneal fat pad (Table 1). The levels of fasting insulin, adiponectin, and free fatty acids were comparable between the strains. SHR.BN16 rats showed substantially reduced concentrations of serum triglycerides and cholesterol (Table 1) compared with SHR rats. The strains showed sharply distinct glucose-concentration time courses during the OGTTs, indicative of markedly improved glucose tolerance in the SHR.BN16 strain compared with the SHR strain. This was reflected by a greater than 50% reduction in the area under the glycemic curve for the SHR.BN16 strain (Table 1, Fig 2).

**Table 1:** Morphometric and metabolic profiles of SHR and SHR.BN16 congenic rats fed a standard diet.

Trait	SHR	SHR.BN16	P_T-test_
N	9	10	
Body weight, g	289 ± 7	266 ± 5	**0.017**
Liver wt., g/100 g b.wt.	2.87 ± 0.04	2.92 ± 0.03	*0*.*37*
Heart wt., g/100 g b.wt.	0.38 ± 0.01	0.38 ± 0.01	*0*.*98*
Kidney wt., g/100 g b.wt.	0.72 ± 0.01	0.70 ± 0.01	*0*.*07*
Adrenals wt., mg/100 g b.wt.	12.3 ± 0.9	15.4 ± 0.3	**0.005**
EFP wt., g/100 g b.wt.	0.84 ± 0.03	0.78 ± 0.02	*0*.*08*
RFP wt., g/100 g b.wt.	1.18 ± 0.05	0.92 ± 0.06	**0.004**
Insulin, nmol/l	0.05 ± 0.01	0.06 ± 0.01	*0*.*68*
AUC_180_, mmol/l/180 min	541 ± 48	255 ± 28	**<0.0001**
FFA, mmol/l	1.41 ± 0.08	1.32 ± 0.08	*0*.*39*
Adiponectin, μg/ml	8.59 ± 0.63	9.60 ± 0.54	*0*.*24*
Triglycerides, mmol/l	0.44 ± 0.01	0.37 ± 0.01	**0.0002**
Total cholesterol, mmol/l	1.41 ± 0.05	1.28 ± 0.03	**0.035**

Values are shown as mean ± S.E.M. The third column shows the significance levels of the pair-wise inter-strain comparisons. b.wt.—body weight; EFP—epididymal fat pad; RFP—retroperitoneal fat pad; FFA—free fatty acids.

### Hemodynamic comparison of the SHR and SHR.BN16 strains

**Fig 3:**
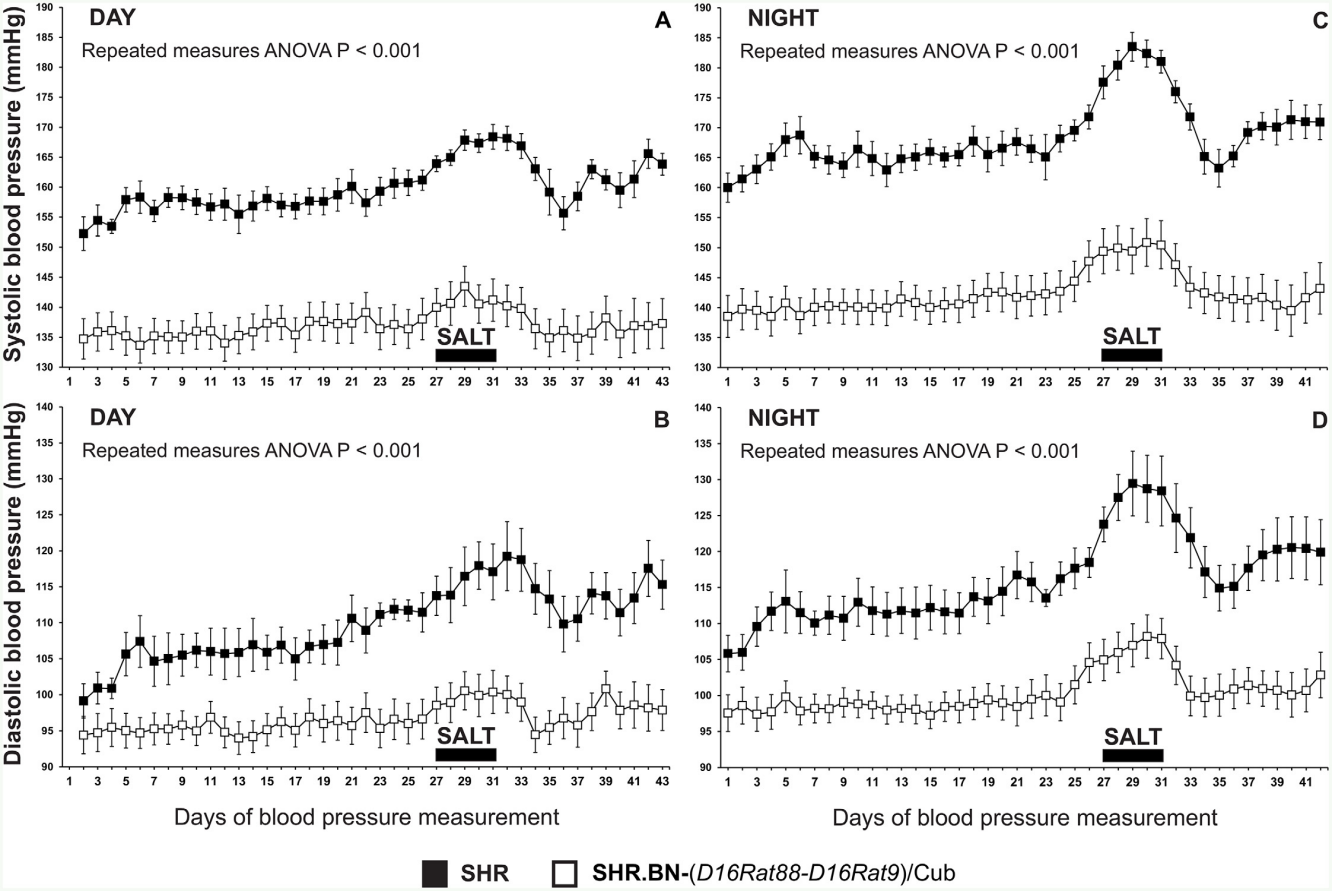
Radiotelemetric blood-pressure assessment in adult male SHR and SHR.BN16 rats. A significant reduction in systolic (Panels A, C) and diastolic (Panels B, D) blood pressure was observed in the SHR.BN-(*D16Rat88-D16Rat9*)/Cub rats during both the daytime (Panels A, B) and the night-time (Panels C, D), as well as under salt load (indicated by a full horizontal bar) (repeated-measures ANOVA, P < 0.001).

Systolic and diastolic blood pressure were both significantly lower in the SHR.BN16 congenic strain than the SHR strain (Fig 3) throughout the entire 41-day period of radiotelemetric blood-pressure measurement (repeated-measures ANOVA, P < 0.001). The high-salt diet caused a significant increase in systolic and diastolic blood pressure in both the SHR and SHR.BN16 strains (P < 0.0001). This was calculated by comparing the systolic and diastolic blood-pressure measurements from the week before administration of the high-salt diet (days 19 to 25) with the 5 day high-salt period (days 28 to 32). The effects of the high-salt diet were more pronounced in the SHR strain than in the SHR.BN16 strain. The SHR strain showed significantly greater increases in daytime systolic blood pressure (P < 0.0005), night-time systolic blood pressure (P < 0.05), and daytime diastolic blood pressure (P < 0.05) than the SHR.BN16 strain. After cessation of the high-salt diet, systolic and diastolic blood pressure in both strains returned to the pre-high-salt values; no significant differences were observed between the mean systolic and diastolic blood-pressure measurements taken before and after administration of the high-salt diet.

### Prioritization of candidate genes

The differential segment of the SHR.BN16 congenic strain harbors 418 annotated genes (NCBI *Rattus norvegicus* Annotation Release 105, Rnor_6.0 assembly). The unaccounted region between the border markers *D16Rat9* (SHR origin) and *D16Rat6* (BN-*Lx* origin) is minute, spanning about 200 kb and containing only three annotated genes (integrator complex subunit 10, *LOC102554585*, and *LOC102554803*). We compared the genomic DNA sequences throughout the SHR.BN16 differential segment between the two parental strains *in silico* [23] to identify variations that were highly conserved between SHR and BN-*Lx*. We identified 44 protein-coding genes within the segment that were predicted to carry nonsynonymous mutations, eight of which were predicted to be probably damaging by the PolyPhen prediction tool (Fig 1, Table 2). There were 1262 identified variations between BN-*Lx* and SHR within the RNO16 segment (S1 Table). It is possible that DNA variations other than the nonsynonymous mutations may be responsible for the observed phenotypic effects. We therefore compared the SHR–BN-*Lx* sequence variations with results from human genome-wide studies. We identified polymorphisms in 19 differential-segment genes that had previously been linked to features of metabolic syndrome in humans (Table 3).

**Table 2:** Gene variants within the SHR.BN16 differential segment predicted to be probably or possibly damaging.

Gene symbol	SNP ID	BN-*Lx* allele	SHR allele	BN-*Lx* AA		SHR AA
*Asb14*	rs197424048	G	**A**	Gly	124	Ser
*Il17rd*	rs197632365	C	**T**	Arg	534	Trp
*Itih1*	rs13447989	A	**G**	Met	816	Thr
*Syt15*	rs105621823	G	**A**	Val	236	Ile
*Ercc6*	rs198154872	G	**A**	Gly	1215	Ser
*RGD1564958*	rs197197423	G	**T**	Gly	10	Cys
*Tmem161a*	rs198810577	C	**T**	Arg	98	His
*Gatad2a*	rs197313667	A	**G**	Ser	235	Gly

DNA variants within the differential segment of the SHR.BN16 congenic strain leading to nonsynonymous mutations predicted to be probably or possibly damaging by the PolyPhen prediction tool. SNP ID—identification of the single nucleotide polymorphism according to the NCBI dbSNP database. AA—the amino acid for BN-*Lx* and SHR strains, together with its position; *Asb14*—ankyrin repeat and SOCS box-containing 14, *Il17rd—*interleukin 17 receptor D, *Itih1—*inter-alpha-trypsin inhibitor heavy chain 1, *Syt15—*synaptotagmin XV, *Ercc6—*excision repair cross-complementing rodent repair deficiency, complementation group 6, *RGD1564958—*similar to glyceraldehyde-3-phosphate dehydrogenase (phosphorylating) (EC 1.2.1.12), *Tmem161a -* transmembrane protein 161A, *Gatad2a*—GATA zinc finger domain containing 2A.

**Table 3:** Genome-wide association studies in human subjects: reported significant associations.

Gene/trait	HSA	Blood pressure	Triglycerides	Cholesterol	Insulin sensitivity	Obesity	Other	SHR/BN-*Lx* variants (exon/intron/other)
***ARHGEF3***	3				Obesity-related glucose increase			14 (1/13/0)
***WNT5A***	3				Type 2 diabetes		Coronary artery calcification	2 (0/1/1)
***CACNA2D3***	3						Coronary artery calcification	99 (0/9/90)
***CACNA1D***	3	SBP, DBP; Hypertension			Insulin resistance			41(8/33/0)
***SFMBT1***	3	Hypertension						6 (0/1/5)
***PBRM1***	3						Adiponectin	4 (1/1/2)
***STAB1***	3			HDL-C		Waist-hip ratio		5 (4/1/0)
***NISCH***	3					Waist-hip ratio		7 (4/3/0)
***BTD***	3						Coronary heart disease	4 (4/0/0)
***ARHGAP22***	10						Diabetic retinopathy	14 (3/9/2)
***GRID1***	10						LV wall thickness; Stearic acid (18:0)	48 (1/3/44)
***NRG3***	10				Insulin resistance; Fasting insulin		Cardiac hypertrophy	67 (1/66/0)
***MYO9B***	19				Glycated hemoglobin			4 (3/1/0)
***COMP***	19	Hypertension (4 studies)						2 (2/0/0)
***CILP2***	19		TG (3 studies)	LDL-C (2 studies)				1 (1/0/0)
***PBX4***	19		TG (3 studies)	LDL-C (2 studies)				3 (0/3/0)
***LPAR2***	19						Sphingolipid levels	1 (1/0/0)
***SLC18A1***	8		TG (3 studies)					1 (1/0/0)
***LPL***	8	TG-BP bivariate	TG (>20 studies)	HDL-C (>20 studies)		Waist circumference	Metabolic syndrome	4 (2/2/0)

Significant associations between features of metabolic syndrome and human genes, for genomic regions syntenic to the SHR.BN16 differential segment and showing sequence variation between the SHR and BN-*Lx* parental strains. Data are based on the Catalog of Published Genome-Wide Association Studies, available at: http://www.ebi.ac.uk/gwas/ (accessed on Nov 26, 2015). HSA—human chromosome, SBP—systolic blood pressure, DBP—diastolic blood pressure, TG—triglycerides, LDL-C—low density lipoprotein cholesterol, HDL-C—high-density lipoprotein cholesterol, LV—left ventricle. *ARHGEF3* -Rho guanine nucleotide exchange factor 3, *WNT5A*—wingless-type MMTV integration site family, member 5A, *CACNA2D3*—calcium channel, voltage-dependent, alpha2/delta subunit 3, *CACNA1D*—calcium channel, voltage-dependent, L type, alpha 1D subunit, *SFMBT1—*Scm-like with four mbt domains 1, *PBRM1*—polybromo 1, *STAB1*—stabilin 1, *NISCH*—nischarin, *BTD*—biotinidase, *SYT15*—synaptotagmin XV, *ARHGAP22*—Rho GTPase activating protein 22, *GRID1*—glutamate receptor, ionotropic, delta 1, *NRG3*—neuregulin 3, *MYO9B* (myosin IXb), *COMP*—cartilage oligomeric matrix protein, *CILP2*—cartilage intermediate layer protein 2, *PBX4*—pre-B-cell leukemia homeobox 4, *LPAR2*—lysophosphatidic acid receptor 2, *SLC18A1*—solute carrier family 18 (vesicular monoamine transporter), member 1, *LPL*—lipoprotein lipase.

## Discussion

The new SHR.BN16 congenic model shows that a limited genomic region of RNO16 in the SHR is important for many features of metabolic syndrome. An existing study with the SHR.BN-(D16Rat87-D16Mgh1)/Jk congenic strain [13] previously demonstrated the involvement of SHR chromosome 16 in hypertension, but the differential segment in the current study was approximately half the size. From the perspective of comparative genomics, the captured region corresponds to segments of murine chromosomes 14 and 8 and to specific regions of human chromosomes 3, 8, 10, and 19 (S1 Fig). The differential segment overlaps with numerous QTLs identified in studies in experimental models that focused on genetic mapping of parameters related to metabolic syndrome (blood pressure QTL nos. 13, 23, 40, 75, 248, 321, 347, and 339; serum cholesterol level QTL no. 9; serum triglyceride QTL no. 23; insulin level QTL nos. 8 and 16; body weight QTL nos. 90 and 95; non-insulin-dependent diabetes mellitus QTL nos. 6 and 29; annotated in the RGD [23]). In addition, over 30 highly significant genome-wide associations with features of metabolic syndrome have been reported in syntenic regions of the human genome, and some of those regions show polymorphisms between the parental strains in the new congenic model (Table 3). As noted above, individual metabolic disturbances have previously been mapped to the region covered by the SHR.BN16 differential segment. However, the current study shows the unique combination of reduced adiposity, lower lipid levels, and improved glucose tolerance due to this limited genomic region.

Several previous studies implicated RNO16 in regulation of blood pressure; however, these studies either assessed blood pressure only under a high-salt diet [12, 28] or observed effects only after salt loading [13]. The distinct results of the current study may stem from several factors, including the age of the animals used (we used 4-month-old rats; most published studies used younger rats), the SHR and BN substrains used, different methods of blood-pressure measurement, and the extent of the differential segment. Here, we demonstrated robust lowering of blood pressure under standard diet conditions with replacement of the smallest SHR-derived RNO16 segment to date, and this effect was further potentiated by salt loading. The amelioration of the lipid profile in the SHR.BN16 congenic strain corroborates the findings from a different RNO16 rat model of metabolic syndrome, the WOKW (Wistar Ottawa Karlsburg W) congenic strain [20]. The biphasic glucose curve in our SHR.BN16 strain is consistent with observations from human studies showing that a biphasic glucose curve is related to more favorable indices for obesity and insulin sensitivity [29]. One limitation of the current study was the selective use of male rats: sex-specific genetic architecture for the traits defining metabolic syndrome has been described both in man [2] and experimental models [30–32].

Information is scarce regarding several of the genes predicted to carry nonsynonymous mutations impairing the function of the protein product, including synaptotagamin XV [33], *Asb14*, *Tmem161a* [34], and *RGD1564958*. Both *Il17rd* and *Itih1*, which codes for one of the heavy chains of the inter-alpha-trypsin protease inhibitor, are expressed in pancreatic beta-cells [35]. Although no mechanism has yet been proposed to directly connect ITIH1 with beta-cell-related functions, interleukin 17 receptor D is a candidate gene for type 1 diabetes and is overexpressed in response to proinflammatory cytokines [36]. A recent large-scale gene-centric meta-analysis of 39 multiethnic type 2 diabetes (T2D) association studies identified a region near the *CILP2* and *GATAD2A* genes as a European T2D risk locus [37]. We identified a single nonsynonymous variant in *Cilp2* (rs197094004 A/G), leading to an amino-acid change from threonine in BN-*Lx* to alanine in SHR (predicted to be benign by PolyPhen). We identified six variations in *Gatad2a*, including two nonsynonymous mutations, one of which is predicted to be damaging. It is not currently possible to fully resolve the key gene(s) behind the alleviation of metabolic syndrome in the SHR.BN16 congenic strain; nevertheless, the genes highlighted in Tables 2 and 3 are prime candidates for future investigation. Although it can be deduced that variations in genes present within the SHR.BN16 differential segment are responsible for the observed phenotypic effects, it is also necessary to consider that their action may be nonlinear in nature. Future studies should therefore seek to identify the specific networks perturbed, including both the variant genes and their interactions with the genomic background. Further research may provide insight into the underlying mechanisms by addressing the functional consequences of the identified DNA-level variations, including *in silico* predictions of the effects of the nonsynonymous mutations on the relevant organ systems and cell types. From among the many possible candidate genes, the most promising ones may be identified by prioritizing approaches that combine prior results with assessment of RNA and protein expression levels, narrowing of the differential segment, and targeted mutagenesis [38].

## Conclusions

Our new congenic model demonstrates that a limited genomic region of RNO16 in the SHR significantly affects many components of metabolic syndrome. Comparison of the DNA sequences in the two parental strains and virtual comparative mapping enabled us to identify genetic variants that are potentially responsible for the observed metabolic and hemodynamic differences between strains. These results may considerably streamline the path towards unveiling of the networks perturbed by these genetic variations, and the role of those networks in the transition to metabolic syndrome.

## Supporting Information

S1 Fig A comparative map of the SHR.BN16 differential segment showing the correspondence between rat chromosome 16 (center) and the syntenic regions of the murine (left) and human (right) genomes. The figure was generated with the Virtual Comparative Map software tool (http://www.animalgenome.org/VCmap).
(PDF)

S1 Table Summary of DNA variations between BN-*Lx* and SHR within the RNO16 segment of SHR.BN16 rat. The table shows complete information on identified DNA variations between BN-*Lx* and SHR rat strains within the RNO16 segment of SHR.BN16 rat using used the Variant Visualizer resource provided by the RGD (http://rgd.mcw.edu/rgdweb/front/select.html) with high conservation settings (0.75–1) determined by PHAST (http://compgen.cshl.edu/phast), minimum read depth set to eight, and exclusion of variants found in fewer than 15% of reads.
(XLSX)
