# Supplementary material for: Isolation of a Genomic Region Affecting Most Components of Metabolic Syndrome in a Chromosome-16 Congenic Rat Model
Source: PLoS One. 2016 Mar 31;11(3):e0152708. doi: 10.1371/journal.pone.0152708 (PMC4816345; doi:10.1371/journal.pone.0152708)
Supplement: S1 Fig — The figure was generated with the Virtual Comparative Map software tool (http://www.animalgenome.org/VCmap). (PDF) [file pone.0152708.s001.pdf]

**S1 Fig. The comparative map of the differential segment in SHR.BN16 congenic rat.**

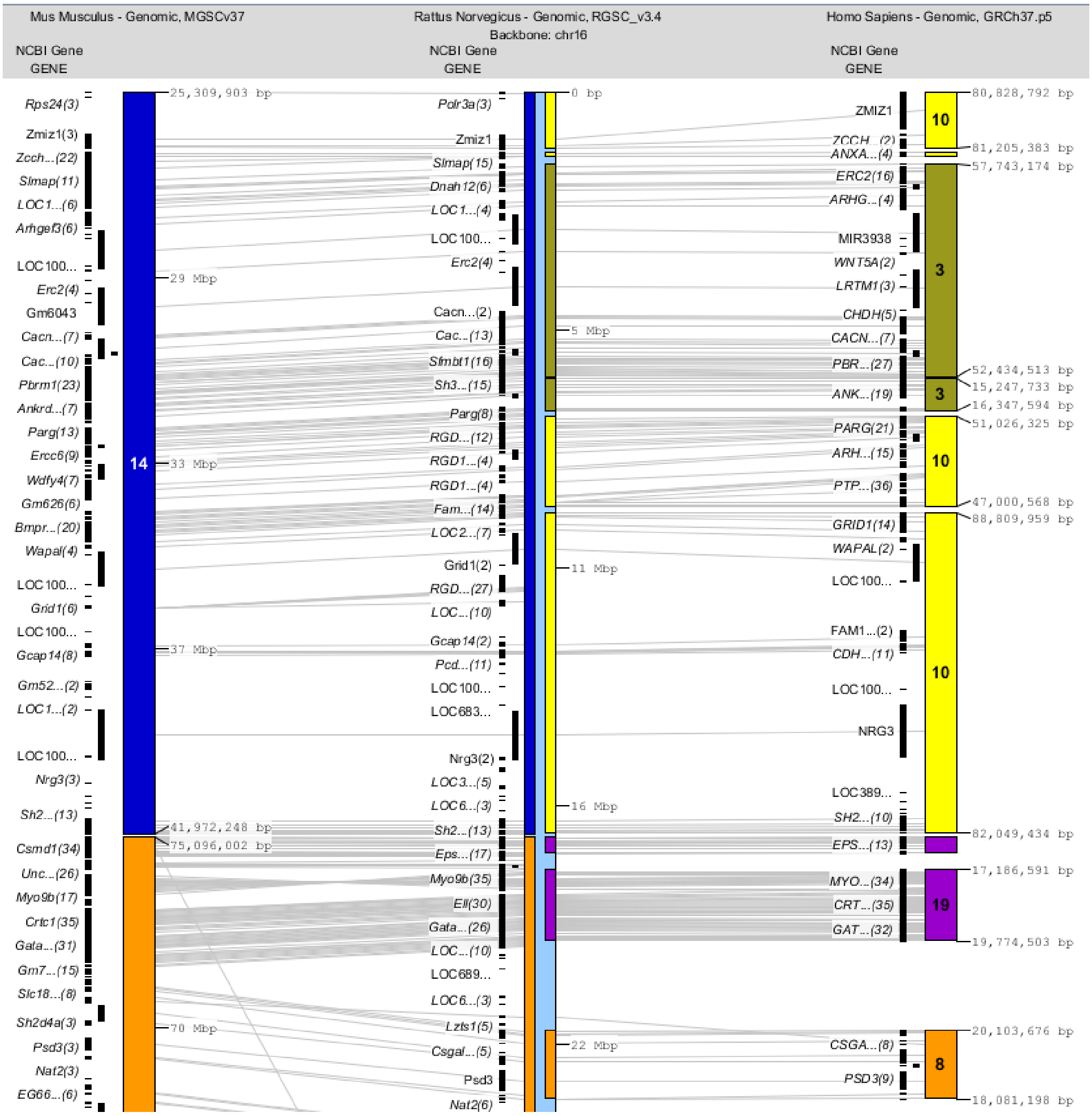


**S1 Fig.** The comparative map of the differential segment in SHR.BN16 congenic rat showing the correspondence between rat chromosome 16 (center) and the syntenic regions of the murine (left) and human (right) genomes. The figure is generated using the Virtual Comparative Map software tool (http://www.animalgenome.org/VCmap/).
